# AI for Anglophone Africa: Unlocking its adoption for responsible solutions in academia-private sector

**DOI:** 10.3389/frai.2023.1133677

**Published:** 2023-04-11

**Authors:** Ramadhani Sinde, Salim Diwani, Judith Leo, Tabu Kondo, Noe Elisa, Jabhera Matogoro

**Affiliations:** ^1^School of Computational and Communication Science and Engineering, Nelson Mandela African Institution of Science and Technology (NM-AIST), Arusha, Tanzania; ^2^Department of Computer Science and Engineering at the College of Informatics and Virtual Education, The University of Dodoma, Dodoma, Tanzania

**Keywords:** Anglophone Africa education, academia-private sector, AI stakeholders, responsible solution, artificial intelligence

## Abstract

In recent years, AI technologies have become indispensable in social and industrial development, yielding revolutionary results in improving labor efficiency, lowering labor costs, optimizing human resource structure, and creating new job demands. To reap the full benefits of responsible AI solutions in Africa, it is critical to investigate existing challenges and propose strategies, policies, and frameworks for overcoming and eliminating them. As a result, this study investigated the challenges of adopting responsible AI solutions in the Academia-Private sectors for Anglophone Africa through literature reviews, expert interviews, and then proposes solutions and framework for the sustainable and successful adoption of responsible AI.

Many African countries are envisioning to achieve the United Nations Sustainable Development Goals (SDGs) target and also become middle-income countries with a semi-industrialized economy by 2030 (Johnston, 2016; Tjoa and Tjoa, 2016; Pedersen, 2018; Vinuesa et al., 2020). Realizing this industrialization target requires basic and advanced skills in emerging technologies, specifically the exploitation of Fourth Industrial Revolution (4IR's) technologies, particularly Artificial Intelligence (AI) and Machine Learning (ML) (Cioffi et al., 2020; Hamdan et al., 2020; Kshetri, 2020; Felice et al., 2022; Noman et al., 2022). Notably, AI has evolved in many phases since its inception to an indispensable role in solving many societal and industrial challenges. Hence, it can significantly impact the Global South by transforming public services delivery in strategic developmental sectors like healthcare, infrastructure, data ecosystem, digital economy, environmental conservation, and agriculture (Benke and Benke, 2018; Heymann et al., 2018; Srivastava, 2018; Nensa et al., 2019; Ifenthaler, 2020; Lopez-Jimenez et al., 2020; Kipkorir-Songol et al., 2021; Kaack et al., 2022). Additionally, AI can provide a means for small and medium-scale industries and businesses to thrive and improve the country's GDP growth rate (Kushwaha and Kar, 2020; Hansen and Bøgh, 2021; Sharma et al., 2022). For instance, in the years 2017, 2018, and 2019, the AI for Good Global Summit organized by ITU, explored different AI solutions that can yield long-term benefits and help achieve the SDGs (Artificial Intelligence ITU for Global Good, 2018; Cioffi et al., 2020; Floridi et al., 2020; Walsh et al., 2020; Holzmeyer, 2021). Such solutions include AI-based prediction of disease outbreaks, AI-enabled drones for power lines inspection, and using AI to help farmers improve crop production with less water, to mention a few (Chen et al., 2019; Jha et al., 2019; Talaviya et al., 2020; Zhang et al., 2020; Abdullahi et al., 2021; Kolozsvari et al., 2021; Mehta et al., 2021; Rovira-Sugranes et al., 2022). Despite AI's enormous promises and opportunities for sustainable development in many other sectors, Anglophone African countries are still not fully prepared to adopt its full benefits (Vinuesa et al., 2020; Wareham, 2021). Lack of preparedness to harness the benefit of responsible AI in the Academia-Private sector has led to failures and challenges to most of the implemented AI-based solutions and interventions that would have solved most of Anglophone Africa's notorious social and economic challenges (Magrabi et al., 2019; Raaijmakers, 2019; Al Mutawa and Rashid, 2020; Briceño, 2020; Chassagnon and Dohan, 2020; Radakovich et al., 2020; Abioye et al., 2021).

Therefore, for the Academia-Private sectors in Anglophone Africa to adopt the full benefits of responsible AI solutions, it is necessary to investigate the existing challenges. Thereafter, establish strategies and mechanisms for overcoming these challenges and lay a foundation for the Anglophone African countries' current and future workforce in AI. Consequently, this study used data collection approaches including online survey and document review. In the review, the study obtained articles published between 2017 and 2022 from the google scholar, science direct, IEEE Xplore and other sources. The search used the following keywords: responsible AI, machine learning (ML), and challenges of AI solutions, and inclusion criteria based on their titles, abstract, and full-text as illustrated in Figure 1. Whereas through the use of PRISMA (Page et al., 2021) approach, a set of queries in the identification stage, the authors identified a total of 646 publications from the database search, then, 231 publications were removed as duplicates, and the remaining 415 were filtered to remove the 307 irrelevant publications based on their titles. Then, the 108 publications were screened to remove 77 publications based on their abstracts. Lastly, the remaining 31 publications were screened based on their full text, and as a result, 21 publications were removed. In the end, only 10 publications were eligible to be included in the review with equal consensus from all authors. Furthermore, forward snowballing process was applied to the 10 eligible articles which were included in the review. Hence, as a result, 4 new eligible articles were added in the review, making a total of reviewed articles to be 14.

**Figure 1:**
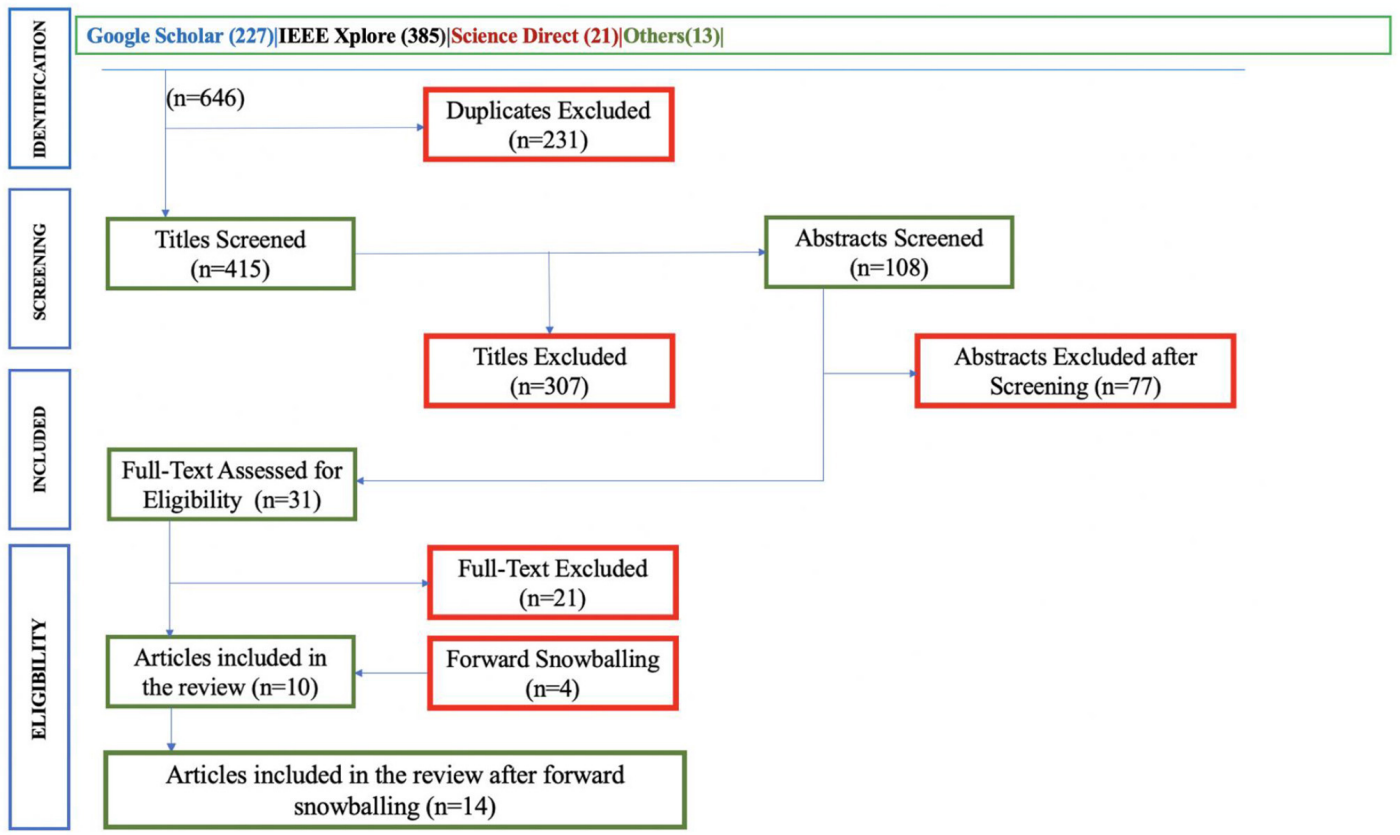
Flow chart showing the selection of publications based on PRISMA.

Furthermore, the expert interview and discussion comprised 44 respondents from Anglophone African countries. The response indicated that thirty-three (75%) were male and eleven (25%) were female, as shown in Figure 2A; four (9.1%) respondents were between 18 to 24 years old, fifteen (34.1%) were between 25 to 34 years old, eighteen (40.9%) were between 35 to 44 years old, and seven (15.9%) were between 45 to 54 years old, as shown in Figure 2C. Whereas, the role of respondents in the AI sector in their current country includes eight (8) academicians, thirteen (13) data scientists, five (5) ICT policy makers, three (3) ICT consultants, five (5) innovators, and ten (10) students. Moreover, the data indicate that the respondents have been in the AI sector as follows, about 82.5% have been in the sector for less than 5 years, about 10% have been in the sector for 5 to 10 years, about 5% have been in the sector for 10 to 15 years, and about 2.5% have been in the sector for more than 15 years as shown in Figure 2B. The study review and expert interview laid the groundwork for proposing the adoption strategy of responsible AI in the Academia-Private sectors for Anglophone Africa.

**Figure 2:**
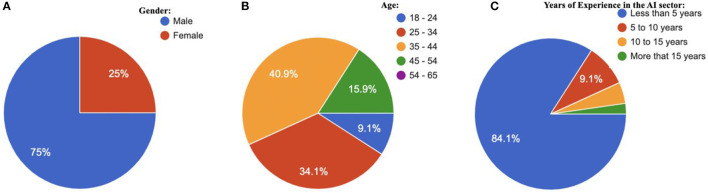
Description of the participants in terms of **(A)** Gender, **(B)** Age, and **(C)** the years of experience in the AI sector.

The general identified and reported challenges (C1-C11) include the following (Schoeman et al., 2017; Alami et al., 2020; Gwagwa et al., 2020a,b, 2021; How et al., 2020; Valle-Cruz et al., 2020; Arakpogun et al., 2021; Sobrino-García, 2021; Amankwah-Amoah and Lu, 2022; López et al., 2022; Zagabathuni and Zagabathuni, 2022; Eke, 2023)^1^:

**C1** Inadequate AI infrastructure for the effective development and long-term implementation of responsible solutions.

**C2** There is a scarcity of African-origin datasets that are relevant, reliable, and of high quality to support research, development, and innovation.

**C3** The majority of existing datasets are still paper-based, and most organizations are unwilling to digitize them.

**C4** Data is underutilized in decision-making and policy-making, even when the data is available.

**C5** Inadequate data management skills and knowledge.

**C6** There are no policies, guidelines, regulations, or strategies in place to facilitate the long-term adoption of AI-based solutions.

**C7** Lack of AI expertise, limited capabilities, and skills to develop appropriate solutions.

**C8** There are no clear specifications or approaches for implementing AI solutions.

**C9** Inconsistent funding for AI-related projects.

**C10** Misconceptions and Lack of Awareness and knowledge about AI Technology.

**C11** There are no clear roadmaps for AI adoption.

Despite the fact that the study was limited to investigating challenges that the Academia-Private sector faces in innovating responsible AI in Anglophone African countries, the recommended adoption strategies and solutions can be applied in other African countries. In general, the major contributions of this study are summarized as follows:

- Proposed strategies, policies and frameworks to overcome and eliminate challenges for responsible AI adoption in Academia-Private sector in Anglophone Africa.
- Brought together each tech spaces, technology hubs and centers in the AI ecosystem.
- Pointed out the cutting-edge insights and research outputs of developing a responsible AI in Anglophone Africa.
- Explored the Academis-Private sector of Responsible AI that drives the economic development of the Anglophone Africa.
- Highlighted the motivation for further development of responsible AI to stimulate Academia-Private sector in Anglophone Africa.

The remainder of this study includes a discussion of the proposed adoption strategies and framework for responsible AI adoption in the Academia-Private sector, as well as a call to action to all AI stakeholders in Anglophone African countries and Africa as a whole.

## Solutions for responsible AI adoption in Anglophone Africa for the academia-private sector

Table 1 categorizes the solutions to the identified challenges for responsible AI adoption into six groups (A1–A6). Each group contains at least one solution (S) that could be used to help the Academia-Private sector in Anglophone Africa adopt responsible AI more smoothly. These solutions will hasten the successful adoption of AI-powered solutions and their application in the Anglophone African Academia-Private sector.

**Table 1:** Solutions for responsible AI adoption.

**Adoption code**	**Category**	**Solution(s)**	**Solution code**
A1	AI leadership and management	Prepare roadmaps to AI adoption	**S1**
		Create and support AI startups	**S2**
		Build-up AI awareness	**S3**
		Create an AI community of practice	**S4**
		Participatory leadership for AI	**S5**
		Develop a cooperating AI ecosystem	**S6**
		International benchmarks	**S7**
A2	AI infrastructure	Create an open AI infrastructure	**S8**
A3	Data management	Data governance	**S9**
A4	AI ethics	Create ethical guidelines and procedures for AI	**S10**
A5	AI policy	Create AI policy	**S11**
A6	Inclusivity	Gender and inclusion in AI	**S12**

**AI Leadership and Management**: Eight solutions fall under this category (i.e., **S1, S2, S3, S4, S5, S6**, and **S7**); **S1**: Academia-Private sector should develop an AI roadmap based on their organizational culture. The roadmap must be tailored to fit the organization's size, maturity level, and proper industry category to ensure desired AI adoption results. **S2**: Academia-Private sector must create and support AI startups through venture investment, funding programs, and linkage with the market. The Academia-Private sector's support should be realized through the growth and scale-up of AI startups. **S3**: Academia-Private sector should provide training and awareness of AI adoption within the community. The capacity building must be accompanied by showcasing practical and proven AI solutions in a particular sector to stimulate interest in AI usage, its adoption, awareness, and increased career competencies. **S4:** Academia-Private sector should support the creation of a sustainable AI Community of Practice (ACP) that shall unite all stakeholders. The ACP should be used as a platform to share resources in the form of training, case studies, and certification to assist companies in making the AI adoption journey easier. **S5**: The academia-Private sector must encourage participatory leadership to involve all staff in the decision-making process. The deployment of AI-powered solutions within organizations is nearly impossible if the management is unaware of the benefits and risks of responsible AI. **S6:** Academia-Private sector should develop a cooperating AI ecosystem comprising various components related to new hardware and software. An AI ecosystem is a collection of AI systems that work together to achieve a common goal. The primary purpose of establishing an AI ecosystem in the Academia-Private sector is to establish a solid and automated data-sharing process between different AI systems. This will allow AI systems to interact with one another in real-time through data sharing. **S7:** Academia-Private sector should perform International Benchmarking by investigating the success indicators in national AI strategies of other successful countries. AI success indicators can be classified as quantitative or qualitative (Heumann, 2018; Martinez-Plumed and Hernandez-Orallo, 2020). Quantitative indicators are observable inputs to AI strategies, such as the budget that must be allocated to AI investment and research funding. Inputs such as new guidelines and regulations for allocating investment and research funding are examples of qualitative indicators. National AI strategies for African countries should include benchmarking and incorporating appropriate success indicators from benchmarked countries. **AI Infrastructure**: This category has one solution (**S8**); **S8:** Academia-Private sector must collaborate to build an open AI infrastructure that all stakeholders can utilize. To facilitate the quick creation of AI solutions, infrastructure should support open standards. A high-level programming interface must be included into the open AI infrastructure to give AI stakeholders the tools they need to create and test AI-powered solutions. **Data Management**: This category has one solution (**S9**); **S9**: Data governance is critical in ensuring that AI produces valuable solutions. It should include all phases of data management strategies such as data identification, collection, preparation, wrangling, validation, annotation, storage, maintenance, usage, and sharing to ensure the data's security. **AI Ethics**: This category has one solution (**S10**); **S10:** Academia-Private sector should develop ethical guidelines and procedures for AI solutions that will govern their design, development and usage. The ethical guidelines and procedures (Kiemde and Kora, 2022)^2^ will be a convenient tool for guiding stakeholders in developing AI-powered products and services with positive effects whilst avoiding negative consequences. As a result, the academic and private sectors must collaborate to create AI-powered solutions that take into account the relationship between accountability, auditability, and explainability of AI-powered products. Finally, in order to keep humans at the forefront of AI adoption and solutions across Africa, the Academia-Private sector must focus more on developing human-centered solutions. **AI Policy**: This category has one solution (**S11**); **S11:** Academia-Private sector must develop AI policy and the terms of use for the developed AI solutions. AI policy is a very important component in AI system implementation. The AI policy will eventually guide management, usage, and sharing of AI-powered products, which is critical to developing high-quality AI-powered products. AI policy includes elements such as ethical purpose and societal benefits, accountability, transparency and explainability, fairness and non-discrimination, safety and reliability, open data and fair competition, privacy, and intellectual property (Nayebare, 2019; Kipkorir-Songol et al., 2021). **Inclusivity**: This category has one solution (**S12**); **S12:** AI systems need to be fair, inclusive, diverse, equitable and representative. For example, the Academia-Private sector should help academic institutions increase the number of girls studying Science, Technology, Engineering, and Math (STEM) (Abe and Chikoko, 2020)^3^ subjects as well as computer science. Other initiatives aimed at youths, marginalized groups, and girls, to name a few, should be implemented to encourage coding and leadership in AI.

## A framework for adoption of responsible AI in Anglophone Africa

**Figure 3:**
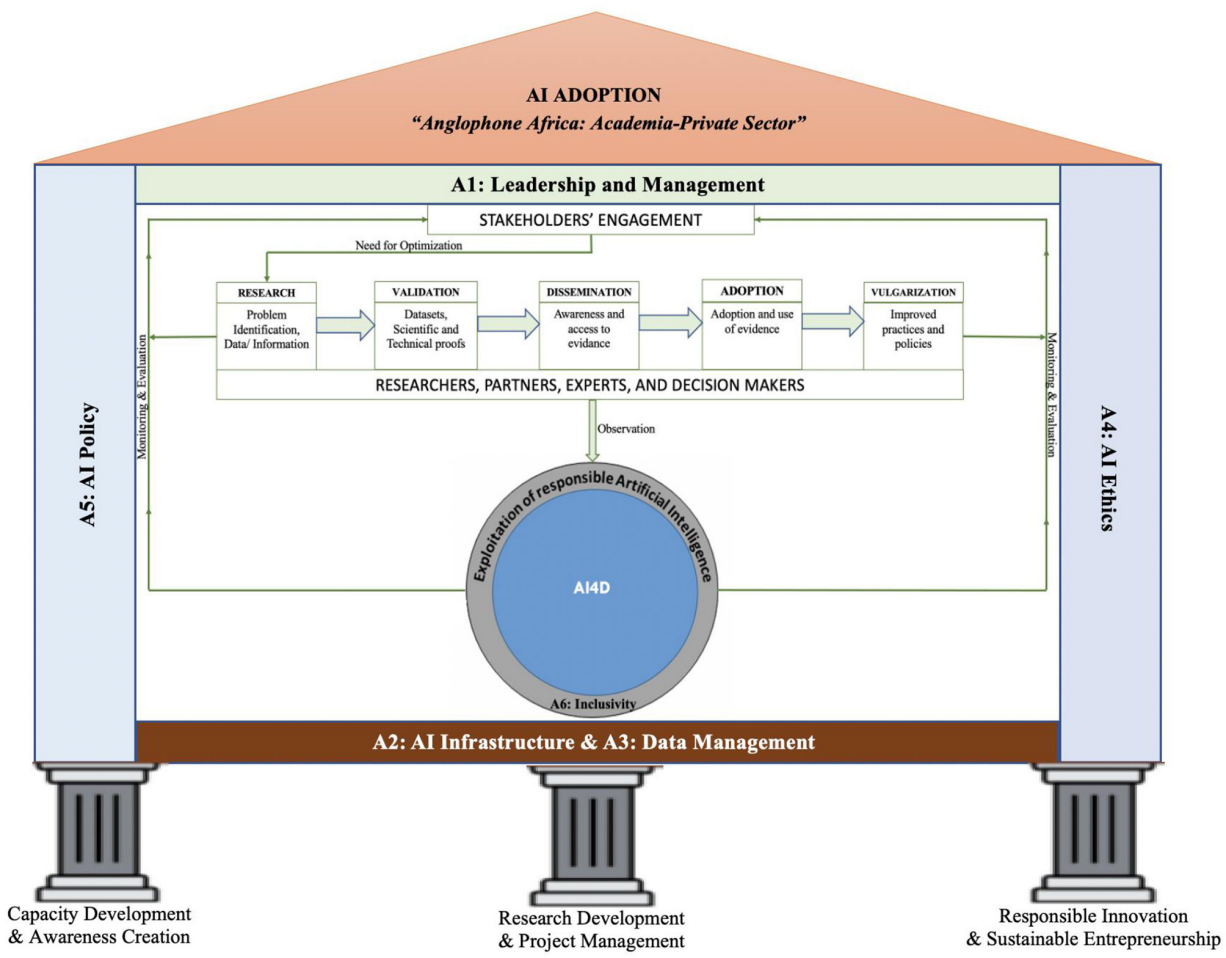
AI adoption framework in Anglophone Africa for academia-private sector.

The study proposes a framework for implementing responsible AI in Anglophone Africa, as shown in Figure 3. The framework is built around three guiding pillars: capacity development and raising awareness; research development and project management; and responsible innovation and sustainable entrepreneurship. All of the adoption strategies and solutions presented in Table 1 are supported by the framework's three pillars. The capacity development and awareness creation pillar focuses on using formal education and on-the-job experience to prepare the Academia-Private sector in Anglophone Africa for responsible AI adoption. The pillar for research development and project management will be realized by supporting responsible AI research, project, and development by local and regional governments in Anglophone Africa. The pillar for responsible innovation and sustainable entrepreneurship is in charge of creating AI-related innovation ecosystems in Anglophone Africa by allocating budget and funding for AI investments. The use of this framework by digital policymakers, technologists, and leaders will strengthen their capacities to face the challenges (technical, legal, social, and ethical) of responsible AI adoption and facilitate the habit of applying appropriate solutions to a specific challenge. Furthermore, this framework will serve as a tool for increasing diversity in AI-related activities and enhancing the adoption and implementation of responsible AI policies by local and regional governments across Anglophone African countries.

## Case studies of AI adoption in Anglophone Africa

There are several case studies regarding the adoption of AI in Anglophone Africa in different domains, including healthcare, education, agriculture, digital economy, infrastructure, and data ecosystem. Some of the case studies include M-SHULE^4^ for education, Computer-Aided Detection for Tuberculosis (CAD4TB) (Murphy et al., 2020) for healthcare, AGROBOT for agriculture,^5^ Kudi^6^ for the digital economy, Artificial Intelligence for Digital Response (AIDR),^7^ and Dr. Elsa Health Assistant technology for healthcare (Development and Challenges, 2019; Intelligence et al., 2020; Proposal and Elsa, 2020).^8^ This study, on the other hand, used Dr. Elsa Health Assistant technology for healthcare as an example to identify some of the challenges to AI adoption in Tanzania.

The Dr. Elsa Health Assistant system employs artificial intelligence (AI) approaches to help medical professionals in rural areas by providing symptom assessment, diagnostic decision support, next step recommendations, and disease outbreak prediction. The Tool is provided to healthcare professionals through a mobile application on a phone or tablet and performs like an experienced pediatrician on a tablet. The smartphone app generates a differential diagnosis and evidence-based recommendations for the patient's next step when the demographics, vital signs, symptoms, and test results of the patient are entered. It is intended to help low-cadre healthcare providers make decisions about their patients through efficient symptom evaluation, strengthening their competence to improve patient outcomes.

An interview with the founder of Dr. Elsa was conducted to identify some of the challenges to AI adoption in Tanzania, and it was revealed that there are some challenges that hinder the Tool's adoption in the country. Some of these challenges include lack of supportive healthcare infrastructure, unavailability of locally located servers and poor network availability; technology literacy and small talent pool; lack of regulatory frameworks; and limited access to high-performance computing. These challenges are briefly discussed as follows:

Lack of supportive health infrastructure, locally located servers and poor network availability: The founder pointed out that, most health facilities, especially public health facilities, do not have digital systems/hardware that we can leverage for AI technology. Either they do not have any technology at all and are relying on paper-based systems, or they have an electronic health records system which utilizes the Government of Tanzania Health Operations Management Information System (GOT-HOMIS) (Thadeus Kissima and Mushi, 2021) or AfyaCare^9^ government systems. These are very difficult to integrate with Dr. Elsa's zool. In addition, network availability is also a problem. That is, the network is unpredictable, especially in remote areas of Tanzania. Furthermore, health data is sensitive and is encouraged to be stored within Tanzanian territory. However, server storage and cloud computing for locally located servers are expensive. An offering from an international cloud provider for $15 per month is more than TZS 800,000 for a local alternative. Also, many hosting providers in Tanzania just resell server space from international providers. Notably, these challenges can be addressed by establishing an open AI infrastructure, as depicted by A2 (S8) in Table 1 and Figure 3.

Technology Literacy and small talent pool: The founder also revealed that, technology literacy is still low in rural Tanzania, and there are still surprising perceptions around AI technology. The founder also reported several misconceptions about AI solutions. Some users were concerned if the founder is linked to freemasons because of entering their data into the system. The founder quotes some positive feedback, “I permit my data to be uploaded into your system since one of my colleagues was cured after using the system”. Additionally, the founder said that “there are many programmers in Tanzania, not as many as we would like, but still enough that we can find programmers when needed”. The biggest problem is finding “engineers who not only know programming language but solve given problems effectively and then come back to optimize for efficiency”. Moreover, “the AI talent pool is very small, and the little that does exist is focused on data-centric machine learning and has little-to-no exposure to other forms of AI”. Notably, these challenges can be tackled through leadership and management, as depicted in A1 (S1 to S7) in Table 1 and Figure 3.

Regulatory Framework: According to the founder, there are a lot of uncertainties because there are vague regulations governing “data” and “data transfer,” and there are no regulations governing algorithmic decision-making. The whole world is still trying to solve these problems, but it just means we are not sure about what the new laws will mean. This challenge can be solved using A5 (S11), as shown in Table 1 and Figure 3.

## Call for action

AI stakeholders are encouraged to develop and deploy solutions equitably and ethically to facilitate the smooth adoption of responsible AI in Anglophone Africa for the Academia-Private sector. As a result, the Academia-Private sector must be proactive in harnessing responsible AI solutions for economic development and investment and establish an effective and sustainable roadmap for innovating responsible AI solutions in Anglophone Africa. In addition, the Academia-Private sector should increase the capacity building of human resources and the enabling infrastructure for AI adoption while recalibrating its laws and regulations regarding AI-friendly policies. Furthermore, the Academia-Private sector should set clear and guiding factors that determine successful, responsible AI innovations, including communication channels, perceived attributes of innovation, type of innovation-decision, nature of the social system, and efforts of change agents. Failure to make these enhancements and tap into the proposed framework and recommended solutions would result in the forced adoption of obsolete and rudimentary AI solutions that would not be adequate for Africa's developmental goals and the needs of its populations.

## Limitations and future works

The challenges and solutions of adopting responsible AI in the Academia-Private sectors for Anglophone Africa presented in this study are limited to the opinions of AI experts and researchers; thus, the general community's opinions are not included in the findings and will remain a piece of future work. Furthermore, the proposed framework for the sustainable and successful adoption of responsible AI is a theoretical solution and also, there is a lack of investigation for equity and inclusivity in the study. Therefore, the study recommends future works to conduct an empirical evaluation of the framework and also investigate equity and inclusion in AI across Anglophone Africa. Despite these limitations and recommendations, the achieved outcomes remain significant to the study area and the collected data.

## Data availability statement

The raw data supporting the conclusions of this article will be made available by the authors, without undue reservation.

## Author contributions

RS, SD, JM, JL, NE, and TK ideated, designed, and coordinated inputs from literature review and other experts in the area to unlock adoption of AI for responsible solutions in Academia-Private sector for Anglophone Africa. JM, RS, and JL wrote introduction, methodology, and challenges for adopting AI in Academia-Private sector. SD, TK, and NE wrote the proposed solutions for adopting AI and explained on how they can be used in one case study for successful adoption of AI in Academia-Private sector for Anglophone Africa. All authors reviewed the paper as final editor.

## Footnotes

^1^WORLD VIEW Look to Africa to advance artificial intelligence.

^2^Ethics and Governance of Artificial Intelligence for Health | OpenWHO. Available online at: https://openwho.org/courses/ethics-ai (accessed August 7, 2022).

^3^Center Africa, S. T. E. M. Available online at: https://www.stemcenter-africa.com/ (accessed August 7, 2022).

^4^M-Shule - Together We Learn, Together We Succeed. Available online at: https://m-shule.com/ (accessed August 7, 2022).

^5^Agrobot: Agricultural Robots. Available online at: https://www.agrobot.com/ (accessed August 7, 2022).

^6^Nomba. Available online at: https://nomba.com/ (accessed August 7, 2022).

^7^AIDR: Artificial Intelligence for Disaster Response. Available online at: http://aidr.qcri.org/ (accessed August 7, 2022.).

^8^Elsa Health | AI for clinical decision support. Available online at: https://www.elsa.health/ (accessed August 7, 2022).

^9^AFYACARE. Available online at: http://www.afyacare.co.id/ (accessed August 7, 2022).
